# Supplementary material for: Proteotranscriptomic Profiling of the Toxic Mucus of Kulikovia alborostrata (Pilidiophora, Nemertea)
Source: Toxins (Basel). 2024 Dec 26;17(1):5. doi: 10.3390/toxins17010005 (PMC11768458; doi:10.3390/toxins17010005)
Supplement: Supplementary file 1 [file toxins-17-00005-s001.zip › Supplementary File S4.zip!/ORF 001200 LINALB/1362d454f90accc2/~ORF001200.html]

Phyre 2 Results for ORF\_001200\_LINALB


|  |  |
| --- | --- |
|  | Retrieve Phyre Job Id |


|  |  |
| --- | --- |
| Email | vas9i-kz@mail.ru |
| Description | ORF\_001200\_LINALB |
| Date | Fri Apr 7 03:17:43 BST 2023 |
| Unique Job ID | 1362d454f90accc2 |
| Sequence | EFLKTQKRCF ... Download FASTA |
| Job Type | **normal** |
| Job Expiry |  |

|  |  |
| --- | --- |
| Summary |  |

Top model

Image coloured by rainbow N → C terminus

|  |  |
| --- | --- |
| Model dimensions (Å): | **X**:24.853 **Y**:14.746 **Z**:16.842 |

|  |  |
| --- | --- |
|  |  |
 Close JSmol | | |

 Model (left) based on template c2f2jA\_ Top template information **PDB header:**antimicrobial protein **Chain:** A: **PDB Molecule:**kalata-b1; **PDBTitle:** solution structure of [w19k, p20n, v21k]-kalata b1 **PDB Entry:** PDBe RCSB PDBj - Confidence and coverage Confidence: \| **81.2%** \| Coverage: \| **47%** 23 residues ( 47% of your sequence) have been modelled with 81.2% confidence by the single highest scoring template. You may wish to submit your sequence to Phyrealarm. This will automatically scan your sequence every week for new potential templates as they appear in the Phyre2 library. Please note: You must be registered and logged in to use Phyrealarm. 3D viewing Interactive 3D view in JSmol For other options to view your downloaded structure offline see the FAQ |

|  |  |
| --- | --- |
| Sequence analysis |  |

|  |  |
| --- | --- |
|  | Download FASTA version |

|  |  |
| --- | --- |
| Secondary structure and disorder prediction [Show] |  |

|  |  |  |  |
| --- | --- | --- | --- |
|  |  |  |  |

|  |  |  |  |  |  |  |  |  |  |  |  |  |  |  |  |  |  |  |  |  |  |  |  |  |  |  |  |  |  |  |  |  |  |  |  |  |  |  |  |  |  |  |  |  |  |  |  |  |  |  |  |  |  |  |  |  |  |  |  |  |  |  |  |  |  |  |  |  |  |  |  |  |  |  |  |  |  |  |  |  |
| --- | --- | --- | --- | --- | --- | --- | --- | --- | --- | --- | --- | --- | --- | --- | --- | --- | --- | --- | --- | --- | --- | --- | --- | --- | --- | --- | --- | --- | --- | --- | --- | --- | --- | --- | --- | --- | --- | --- | --- | --- | --- | --- | --- | --- | --- | --- | --- | --- | --- | --- | --- | --- | --- | --- | --- | --- | --- | --- | --- | --- | --- | --- | --- | --- | --- | --- | --- | --- | --- | --- | --- | --- | --- | --- | --- | --- | --- | --- | --- | --- |
|  |  | 1 | . | . | . | . | . | . | . | . | 10 | . | . | . | . | . | . | . | . | . | 20 | . | . | . | . | . | . | . | . | . | 30 | . | . | . | . | . | . | . | . | . | 40 | . | . | . | . | . | . | . | . | . |  |  |  |  |  |  |  |  |  |  |  |  |  |  |  |  |  |  |  |  |  |  |  |  |  |  |  |  |  |  |
| Sequence |  | E | F | L | K | T | Q | K | R | C | F | S | R | G | E | G | C | A | F | G | G | T | C | C | Y | G | S | M | C | S | W | F | K | C | I | R | N | P | K | Q | P | I | Y | Y | E | T | T | Y | T | N |  |  |  |  |  |  |  |  |  |  |  |  |  |  |  |  |  |  |  |  |  |  |  |  |  |  |  |  |  |  |
| Secondary structure |  | --- | --- | --- |  |  |  |  |  |  |  |  | --- | --- | --- | --- |  |  | --- | --- | --- |  |  |  |  | --- |  |  |  |  |  |  |  |  |  |  | --- | --- | --- | --- | --- | --- |  |  |  |  |  |  | --- | --- |  |  |  |  |  |  |  |  |  |  |  |  |  |  |  |  |  |  |  |  |  |  |  |  |  |  |  |  |  |  |
| SS confidence |  | --- | --- | --- | --- | --- | --- | --- | --- | --- | --- | --- | --- | --- | --- | --- | --- | --- | --- | --- | --- | --- | --- | --- | --- | --- | --- | --- | --- | --- | --- | --- | --- | --- | --- | --- | --- | --- | --- | --- | --- | --- | --- | --- | --- | --- | --- | --- | --- | --- |  |  |  |  |  |  |  |  |  |  |  |  |  |  |  |  |  |  |  |  |  |  |  |  |  |  |  |  |  |  |
| Disorder |  | **?** | **?** | **?** | **?** | **?** | **?** | **?** | **?** | **?** | **?** | **?** | **?** | **?** | **?** | **?** | **?** | **?** | **?** | **?** | **?** | **?** | **?** | **?** | **?** | **?** | **?** | **?** | **?** | **?** | **?** | **?** | **?** | **?** | **?** | **?** | **?** | **?** | **?** | **?** | **?** | **?** | **?** | **?** | **?** | **?** | **?** | **?** | **?** | **?** |  |  |  |  |  |  |  |  |  |  |  |  |  |  |  |  |  |  |  |  |  |  |  |  |  |  |  |  |  |  |
| Disorder confidence |  | --- | --- | --- | --- | --- | --- | --- | --- | --- | --- | --- | --- | --- | --- | --- | --- | --- | --- | --- | --- | --- | --- | --- | --- | --- | --- | --- | --- | --- | --- | --- | --- | --- | --- | --- | --- | --- | --- | --- | --- | --- | --- | --- | --- | --- | --- | --- | --- | --- |  |  |  |  |  |  |  |  |  |  |  |  |  |  |  |  |  |  |  |  |  |  |  |  |  |  |  |  |  |  |
|  | | | | | | | | | | | | | | | | | | | | | | | | | | | | | | | | | | | | | | | | | | | | | | | | | | | | | | | | | | | | | | | | | | | | | | | | | | | | | | | | |

|  |  |  |  |  |  |  |  |  |  |  |  |
| --- | --- | --- | --- | --- | --- | --- | --- | --- | --- | --- | --- |
| Confidence Key | | | | | | | | | | | |
| High(9) |  |  |  |  |  |  |  |  |  |  | Low (0) |

|  |  |
| --- | --- |
| **?** | Disordered ( 20%) |
|  | Alpha helix ( 37%) |
|  |  |

Beta strand ( 24%)

|  |  |
| --- | --- |
| Domain analysis [Show] |  |

|  |  |  |  |
| --- | --- | --- | --- |
|  |  |  |  |

|  |
| --- |
| (Rank \| Aligned region) |
| (1 \| (--- \| c2f2jA\_ \| ---); PDB 2f2j chain A **Region:** 13 - 36 **Aligned:** 23 **Modelled:** 24 **Confidence:** 81.2% **Identity:** 61% **PDB header:**antimicrobial protein **Chain:** A: **PDB Molecule:**kalata-b1; **PDBTitle:** solution structure of [w19k, p20n, v21k]-kalata b1 **PDB Entry:** PDBe RCSB PDBj Phyre2; 2 \| (--- \| d1n1ua\_ \| ---); PDB 1n1u chain A **Region:** 13 - 36 **Aligned:** 23 **Modelled:** 24 **Confidence:** 67.2% **Identity:** 61% **Fold:** Knottins (small inhibitors, toxins, lectins) **Superfamily:** Cyclotides **Family:** Kalata B1 Phyre2; 3 \| (--- \| c1n1uA\_ \| ---); PDB 1n1u chain A **Region:** 13 - 36 **Aligned:** 23 **Modelled:** 24 **Confidence:** 67.2% **Identity:** 61% **PDB header:**antibiotic **Chain:** A: **PDB Molecule:**kalata b1; **PDBTitle:** nmr structure of [ala1,15]kalata b1 **PDB Entry:** PDBe RCSB PDBj Phyre2; 4 \| (--- \| c7k7xA\_ \| ---); PDB 7k7x chain A **Region:** 13 - 36 **Aligned:** 23 **Modelled:** 24 **Confidence:** 62.5% **Identity:** 61% **PDB header:**immunosuppressant **Chain:** A: **PDB Molecule:**cyclotide pase a; **PDBTitle:** solution structure of the cyclotide pase a **PDB Entry:** PDBe RCSB PDBj Phyre2; 5 \| (--- \| c4ttoA\_ \| ---); PDB 4tto chain A **Region:** 13 - 36 **Aligned:** 23 **Modelled:** 23 **Confidence:** 60.5% **Identity:** 61% **PDB header:**plant protein **Chain:** A: **PDB Molecule:**kalata-b1; **PDBTitle:** quasi-racemic structure of [v25a] kalata b1 **PDB Entry:** PDBe RCSB PDBj Phyre2; 6 \| (--- \| c4ttmA\_ \| ---); PDB 4ttm chain A **Region:** 13 - 36 **Aligned:** 23 **Modelled:** 23 **Confidence:** 59.2% **Identity:** 61% **PDB header:**plant protein **Chain:** A: **PDB Molecule:**kalata-b1; **PDBTitle:** racemic structure of kalata b1 (kb1) **PDB Entry:** PDBe RCSB PDBj Phyre2; 7 \| (--- \| d1nb1a\_ \| ---); PDB 1nb1 chain A **Region:** 13 - 36 **Aligned:** 23 **Modelled:** 24 **Confidence:** 59.0% **Identity:** 61% **Fold:** Knottins (small inhibitors, toxins, lectins) **Superfamily:** Cyclotides **Family:** Kalata B1 Phyre2; 8 \| (--- \| c1nb1A\_ \| ---); PDB 1nb1 chain A **Region:** 13 - 36 **Aligned:** 23 **Modelled:** 24 **Confidence:** 59.0% **Identity:** 61% **PDB header:**antibiotic **Chain:** A: **PDB Molecule:**kalata b1; **PDBTitle:** high resolution solution structure of kalata b1 **PDB Entry:** PDBe RCSB PDBj Phyre2; 9 \| (--- \| c2khbA\_ \| ---); PDB 2khb chain A **Region:** 13 - 36 **Aligned:** 23 **Modelled:** 23 **Confidence:** 58.9% **Identity:** 61% **PDB header:**antimicrobial protein **Chain:** A: **PDB Molecule:**kalata-b1; **PDBTitle:** solution structure of linear kalata b1 (loop 6) **PDB Entry:** PDBe RCSB PDBj Phyre2; 10 \| (--- \| c7lhcA\_ \| ---); PDB 7lhc chain A **Region:** 13 - 36 **Aligned:** 23 **Modelled:** 23 **Confidence:** 58.0% **Identity:** 61% **PDB header:**plant protein **Chain:** A: **PDB Molecule:**kalata-b1; **PDBTitle:** nmr solution structure of [t20k]kalata b1 **PDB Entry:** PDBe RCSB PDBj Phyre2; 11 \| (--- \| c2mn1A\_ \| ---); PDB 2mn1 chain A **Region:** 13 - 36 **Aligned:** 23 **Modelled:** 24 **Confidence:** 37.7% **Identity:** 61% **PDB header:**unknown function **Chain:** A: **PDB Molecule:**kalata b1[w23ww]; **PDBTitle:** solution structure of kalata b1[w23ww] **PDB Entry:** PDBe RCSB PDBj Phyre2; 12 \| (--- \| c4ttnA\_ \| ---); PDB 4ttn chain A **Region:** 13 - 36 **Aligned:** 23 **Modelled:** 24 **Confidence:** 57.8% **Identity:** 57% **PDB header:**plant protein **Chain:** A: **PDB Molecule:**kalata-b1; **PDBTitle:** quasi-racemic structure of [g6a]kalata b1 **PDB Entry:** PDBe RCSB PDBj Phyre2; 13 \| (--- \| d1pt4a\_ \| ---); PDB 1pt4 chain A **Region:** 13 - 36 **Aligned:** 23 **Modelled:** 24 **Confidence:** 57.5% **Identity:** 57% **Fold:** Knottins (small inhibitors, toxins, lectins) **Superfamily:** Cyclotides **Family:** Kalata B1 Phyre2; 14 \| (--- \| c2kukA\_ \| ---); PDB 2kuk chain A **Region:** 13 - 36 **Aligned:** 23 **Modelled:** 24 **Confidence:** 39.3% **Identity:** 57% **PDB header:**antiviral protein **Chain:** A: **PDB Molecule:**leaf cyclotide 2; **PDBTitle:** solution structure of vhl-2 **PDB Entry:** PDBe RCSB PDBj Phyre2; 15 \| (--- \| c7rfaA\_ \| ---); PDB 7rfa chain A **Region:** 13 - 35 **Aligned:** 22 **Modelled:** 23 **Confidence:** 35.9% **Identity:** 59% **PDB header:**biosynthetic protein **Chain:** A: **PDB Molecule:**kalata-b4; **PDBTitle:** nmr solution structure of linear [t20k]kalatab1 **PDB Entry:** PDBe RCSB PDBj Phyre2; 16 \| (--- \| c1jjzA\_ \| ---); PDB 1jjz chain A **Region:** 13 - 35 **Aligned:** 22 **Modelled:** 23 **Confidence:** 35.3% **Identity:** 59% **PDB header:**plant protein **Chain:** A: **PDB Molecule:**kalata-b4; **PDBTitle:** refined structure and disulfide pairing of the kalata b1 peptide **PDB Entry:** PDBe RCSB PDBj Phyre2; 17 \| (--- \| c1k48A\_ \| ---); PDB 1k48 chain A **Region:** 13 - 35 **Aligned:** 22 **Modelled:** 23 **Confidence:** 33.4% **Identity:** 59% **PDB header:**plant protein **Chain:** A: **PDB Molecule:**kalata b1; **PDBTitle:** refined structure and disulfide pairing of the kalata b1 peptide **PDB Entry:** PDBe RCSB PDBj Phyre2; 18 \| (--- \| c2f2iA\_ \| ---); PDB 2f2i chain A **Region:** 18 - 36 **Aligned:** 19 **Modelled:** 19 **Confidence:** 50.1% **Identity:** 63% **PDB header:**antimicrobial protein **Chain:** A: **PDB Molecule:**kalata-b1; **PDBTitle:** solution structure of [p20d,v21k]-kalata b1 **PDB Entry:** PDBe RCSB PDBj Phyre2; 19 \| (--- \| c2jwmA\_ \| ---); PDB 2jwm chain A **Region:** 13 - 36 **Aligned:** 24 **Modelled:** 24 **Confidence:** 30.0% **Identity:** 46% **PDB header:**plant protein **Chain:** A: **PDB Molecule:**kalata-b7; **PDBTitle:** nmr spatial srtucture of ternary complex kalata b7/mn2+/dpc micelle **PDB Entry:** PDBe RCSB PDBj Phyre2; 20 \| (--- \| c3e4hA\_ \| ---); PDB 3e4h chain A **Region:** 13 - 36 **Aligned:** 24 **Modelled:** 24 **Confidence:** 15.6% **Identity:** 46% **PDB header:**plant protein **Chain:** A: **PDB Molecule:**varv peptide f; **PDBTitle:** crystal structure of the cyclotide varv f **PDB Entry:** PDBe RCSB PDBj Phyre2; 21 \| (--- \| --- \| ---)) |

|  |  |
| --- | --- |
| Detailed template information [Hide] |  |

|  |  |  |  |
| --- | --- | --- | --- |
|  |  |  |  |

| # | Template | Alignment Coverage | 3D Model | Confidence | % i.d. | Template Information |
| --- | --- | --- | --- | --- | --- | --- |

|  |  |  |  |  |  |  |
| --- | --- | --- | --- | --- | --- | --- |
| 1 | c2f2jA\_ | --- \| --- \| --- |  | 81.2 | 61 |


 (**PDB header:**antimicrobial protein **Chain:** A: **PDB Molecule:**kalata-b1; **PDBTitle:** solution structure of [w19k, p20n, v21k]-kalata b1 **PDB Entry:** PDBe RCSB PDBj) |
| 2 | d1n1ua\_ | --- \| --- \| --- |  | 67.2 | 61 | **Fold:** Knottins (small inhibitors, toxins, lectins) **Superfamily:** Cyclotides **Family:** Kalata B1 **PDB entry:** PDBe RCSB PDBj |
| 3 | c1n1uA\_ | --- \| --- \| --- |  | 67.2 | 61 |


 (**PDB header:**antibiotic **Chain:** A: **PDB Molecule:**kalata b1; **PDBTitle:** nmr structure of [ala1,15]kalata b1 **PDB Entry:** PDBe RCSB PDBj) |
| 4 | c7k7xA\_ | --- \| --- \| --- |  | 62.5 | 61 |


 (**PDB header:**immunosuppressant **Chain:** A: **PDB Molecule:**cyclotide pase a; **PDBTitle:** solution structure of the cyclotide pase a **PDB Entry:** PDBe RCSB PDBj) |
| 5 | c4ttoA\_ | --- \| --- \| --- |  | 60.5 | 61 |


 (**PDB header:**plant protein **Chain:** A: **PDB Molecule:**kalata-b1; **PDBTitle:** quasi-racemic structure of [v25a] kalata b1 **PDB Entry:** PDBe RCSB PDBj) |
| 6 | c4ttmA\_ | --- \| --- \| --- |  | 59.2 | 61 |


 (**PDB header:**plant protein **Chain:** A: **PDB Molecule:**kalata-b1; **PDBTitle:** racemic structure of kalata b1 (kb1) **PDB Entry:** PDBe RCSB PDBj) |
| 7 | d1nb1a\_ | --- \| --- \| --- |  | 59.0 | 61 | **Fold:** Knottins (small inhibitors, toxins, lectins) **Superfamily:** Cyclotides **Family:** Kalata B1 **PDB entry:** PDBe RCSB PDBj |
| 8 | c1nb1A\_ | --- \| --- \| --- |  | 59.0 | 61 |


 (**PDB header:**antibiotic **Chain:** A: **PDB Molecule:**kalata b1; **PDBTitle:** high resolution solution structure of kalata b1 **PDB Entry:** PDBe RCSB PDBj) |
| 9 | c2khbA\_ | --- \| --- \| --- |  | 58.9 | 61 |


 (**PDB header:**antimicrobial protein **Chain:** A: **PDB Molecule:**kalata-b1; **PDBTitle:** solution structure of linear kalata b1 (loop 6) **PDB Entry:** PDBe RCSB PDBj) |
| 10 | c7lhcA\_ | --- \| --- \| --- |  | 58.0 | 61 |


 (**PDB header:**plant protein **Chain:** A: **PDB Molecule:**kalata-b1; **PDBTitle:** nmr solution structure of [t20k]kalata b1 **PDB Entry:** PDBe RCSB PDBj) |
| 11 | c2mn1A\_ | --- \| --- \| --- |  | 37.7 | 61 |


 (**PDB header:**unknown function **Chain:** A: **PDB Molecule:**kalata b1[w23ww]; **PDBTitle:** solution structure of kalata b1[w23ww] **PDB Entry:** PDBe RCSB PDBj) |
| 12 | c4ttnA\_ | --- \| --- \| --- |  | 57.8 | 57 |


 (**PDB header:**plant protein **Chain:** A: **PDB Molecule:**kalata-b1; **PDBTitle:** quasi-racemic structure of [g6a]kalata b1 **PDB Entry:** PDBe RCSB PDBj) |
| 13 | d1pt4a\_ | --- \| --- \| --- |  | 57.5 | 57 | **Fold:** Knottins (small inhibitors, toxins, lectins) **Superfamily:** Cyclotides **Family:** Kalata B1 **PDB entry:** PDBe RCSB PDBj |
| 14 | c2kukA\_ | --- \| --- \| --- |  | 39.3 | 57 |


 (**PDB header:**antiviral protein **Chain:** A: **PDB Molecule:**leaf cyclotide 2; **PDBTitle:** solution structure of vhl-2 **PDB Entry:** PDBe RCSB PDBj) |
| 15 | c7rfaA\_ | --- \| --- \| --- |  | 35.9 | 59 |


 (**PDB header:**biosynthetic protein **Chain:** A: **PDB Molecule:**kalata-b4; **PDBTitle:** nmr solution structure of linear [t20k]kalatab1 **PDB Entry:** PDBe RCSB PDBj) |
| 16 | c1jjzA\_ | --- \| --- \| --- |  | 35.3 | 59 |


 (**PDB header:**plant protein **Chain:** A: **PDB Molecule:**kalata-b4; **PDBTitle:** refined structure and disulfide pairing of the kalata b1 peptide **PDB Entry:** PDBe RCSB PDBj) |
| 17 | c1k48A\_ | --- \| --- \| --- |  | 33.4 | 59 |


 (**PDB header:**plant protein **Chain:** A: **PDB Molecule:**kalata b1; **PDBTitle:** refined structure and disulfide pairing of the kalata b1 peptide **PDB Entry:** PDBe RCSB PDBj) |
| 18 | c2f2iA\_ | --- \| --- \| --- |  | 50.1 | 63 |


 (**PDB header:**antimicrobial protein **Chain:** A: **PDB Molecule:**kalata-b1; **PDBTitle:** solution structure of [p20d,v21k]-kalata b1 **PDB Entry:** PDBe RCSB PDBj) |
| 19 | c2jwmA\_ | --- \| --- \| --- |  | 30.0 | 46 |


 (**PDB header:**plant protein **Chain:** A: **PDB Molecule:**kalata-b7; **PDBTitle:** nmr spatial srtucture of ternary complex kalata b7/mn2+/dpc micelle **PDB Entry:** PDBe RCSB PDBj) |
| 20 | c3e4hA\_ | --- \| --- \| --- |  | 15.6 | 46 |


 (**PDB header:**plant protein **Chain:** A: **PDB Molecule:**varv peptide f; **PDBTitle:** crystal structure of the cyclotide varv f **PDB Entry:** PDBe RCSB PDBj) |
| 21 | c2m9oA\_ | --- \| --- \| --- | not modelled | 15.4 | 46 |


 (**PDB header:**plant protein **Chain:** A: **PDB Molecule:**kalata-b7; **PDBTitle:** solution structure of kalata b7 **PDB Entry:** PDBe RCSB PDBj) |


|  |  |
| --- | --- |
| Binding site prediction |  |

The confidence in the top model (c2f2jA\_ 81.2% confidence) was considered too low (<90%) for submission to 3DLigandSite

You may manually submit your model to 3DLigandSite, but please be aware that results may be misleading

---

Phyre is now FREE for commercial users!

All images and data generated by Phyre2 are free to use in any
publication with acknowledgement

**Please cite:** The Phyre2 web portal for protein modeling, prediction and analysis.

Kelley LA *et al.*. *Nature Protocols*
10, 845-858 (2015)[pdf]
[Citation
link]

If you use the binding site
predictions from 3DLigandSite, **please also cite:**

3DLigandSite: predicting ligand-binding sites using similar structures.

Wass MN, Kelley LA and Sternberg
MJ *Nucleic Acids Research* 38, W469-73 (2010) [PubMed]

© Structural Bioinformatics Group

Imperial College London

Lawrence
Kelley, Michael Sternberg

Disclaimer

Terms and Conditions

|  |
| --- |
| **Component software** |
| Template detection: HHpred 1.51 |
| Secondary structure prediction: Psi-pred 2.5 |
| Disorder prediction: Disopred 2.4 |
| Transmembrane prediction: Memsat\_SVM |
| Multi-template modelling and *ab initio*: Poing 1.0 |
